# Supplementary figures and images for: A new simple brain segmentation method for extracerebral intracranial tumors
Source: PLoS One. 2020 Apr 17;15(4):e0230754. doi: 10.1371/journal.pone.0230754 (PMC7164623; doi:10.1371/journal.pone.0230754)

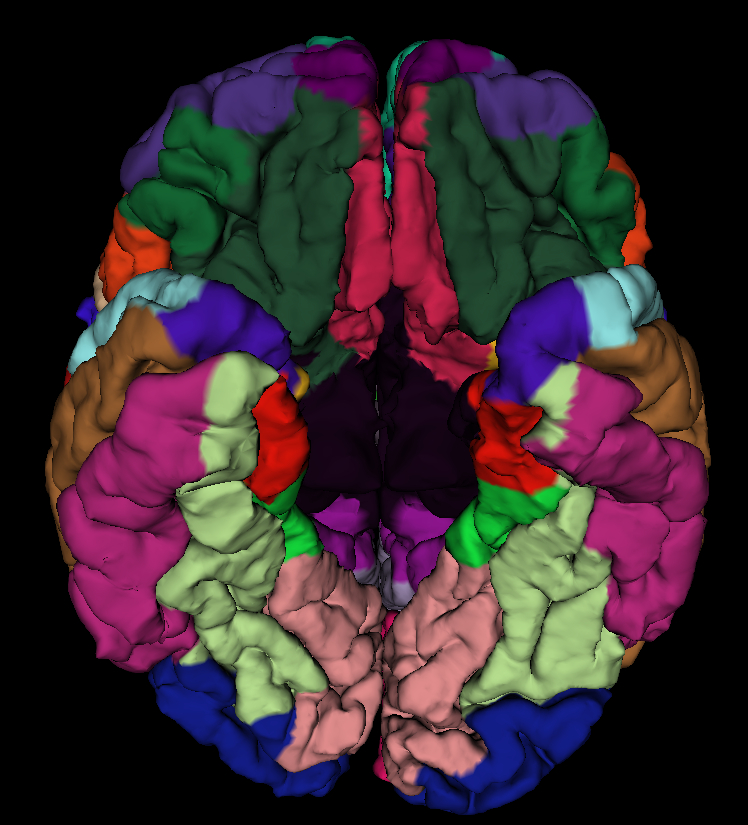

Supplement: S1 File — (ZIP) [file pone.0230754.s001.zip › SUB6/picture/color brain/1.jpg]

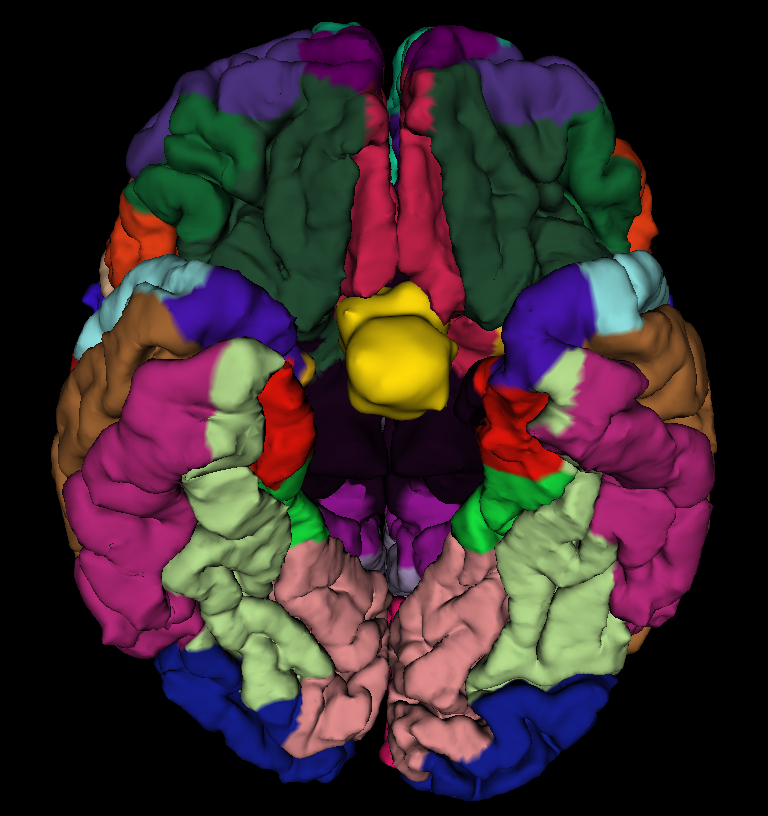

Supplement: S1 File — (ZIP) [file pone.0230754.s001.zip › SUB6/picture/color brain/2.jpg]

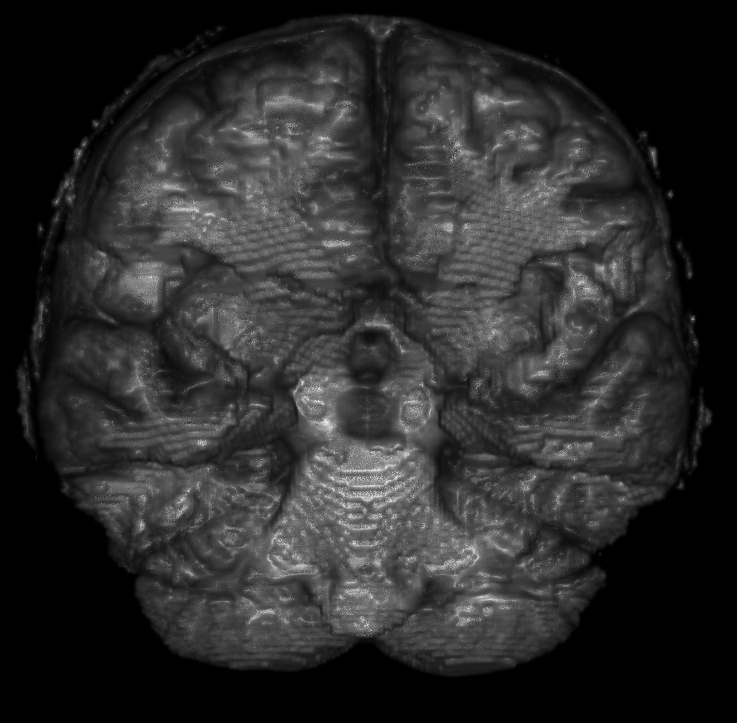

Supplement: S1 File — (ZIP) [file pone.0230754.s001.zip › SUB6/picture/VR/1.jpg]

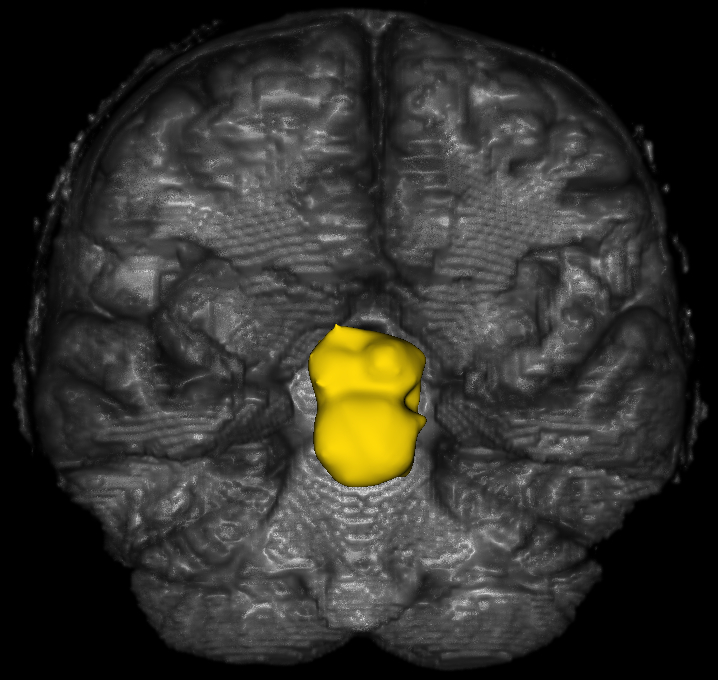

Supplement: S1 File — (ZIP) [file pone.0230754.s001.zip › SUB6/picture/VR/2.jpg]

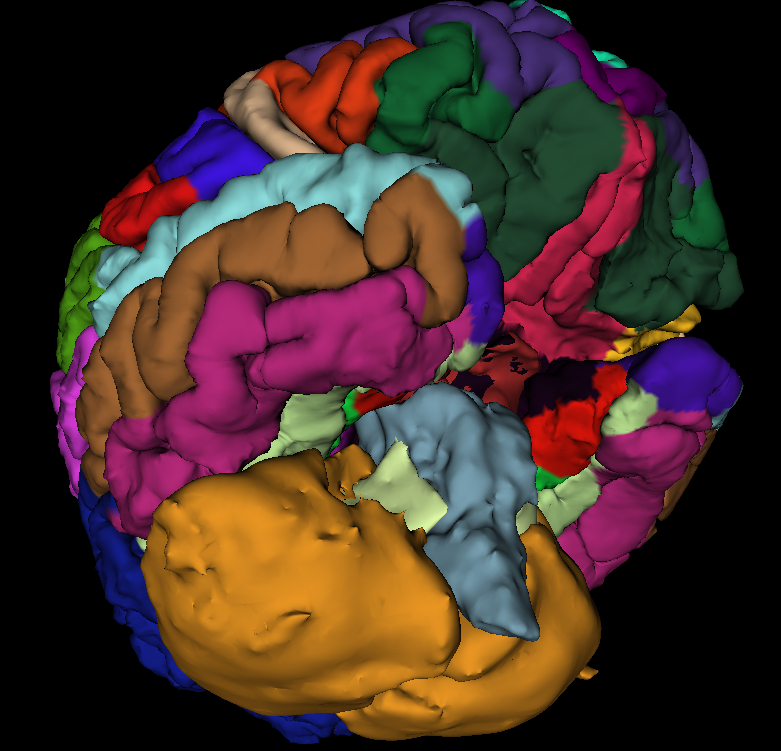

Supplement: S2 File — (ZIP) [file pone.0230754.s002.zip › SUB8/picture/color brain/1.jpg]

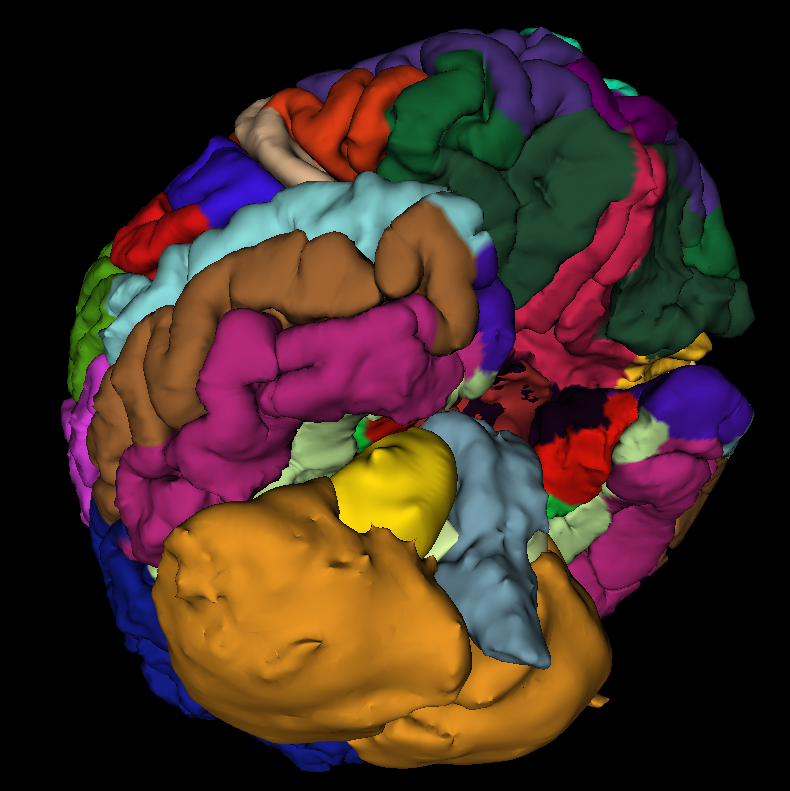

Supplement: S2 File — (ZIP) [file pone.0230754.s002.zip › SUB8/picture/color brain/2.jpg]

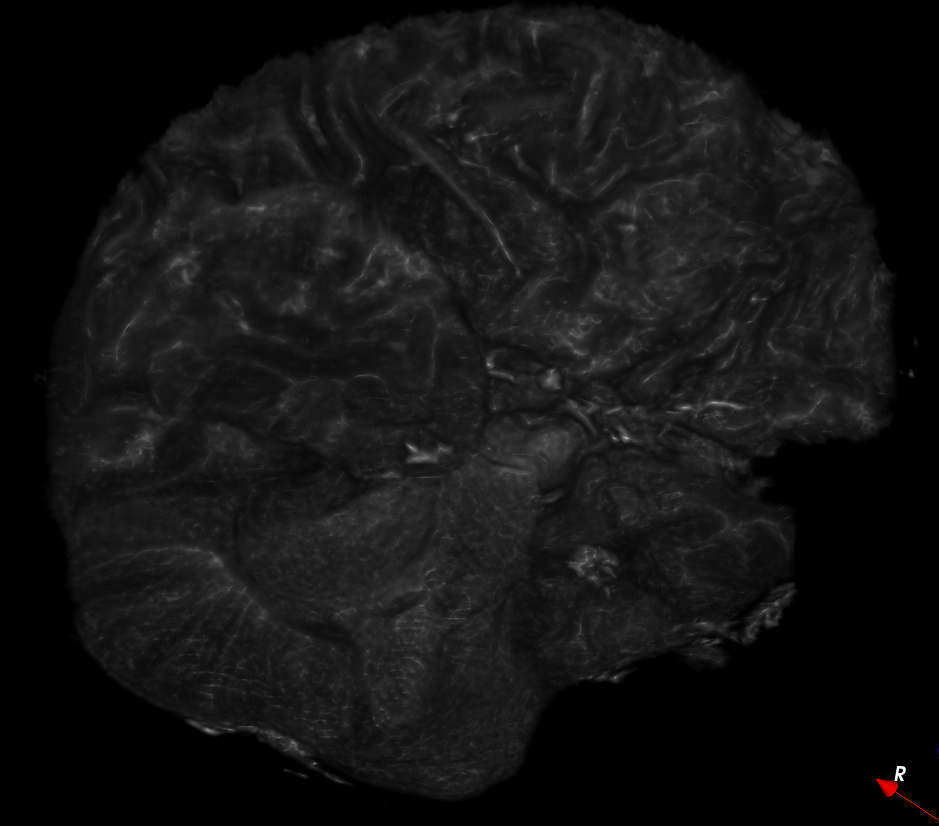

Supplement: S2 File — (ZIP) [file pone.0230754.s002.zip › SUB8/picture/VR/1.jpg]

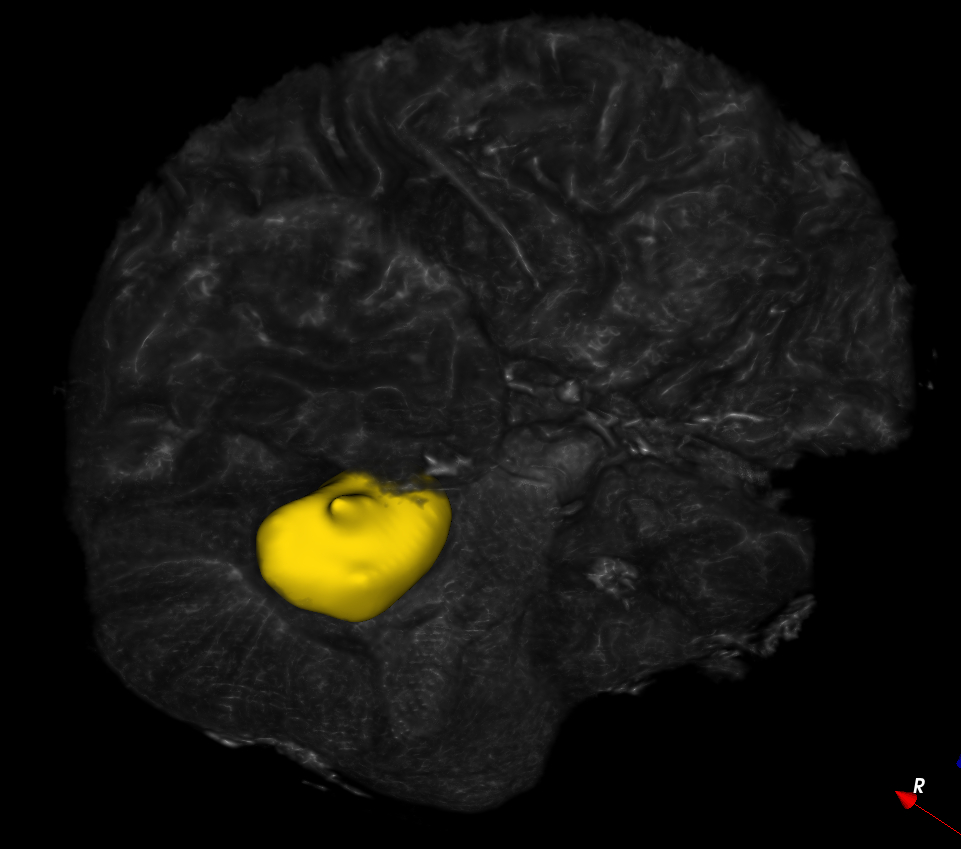

Supplement: S2 File — (ZIP) [file pone.0230754.s002.zip › SUB8/picture/VR/2.jpg]

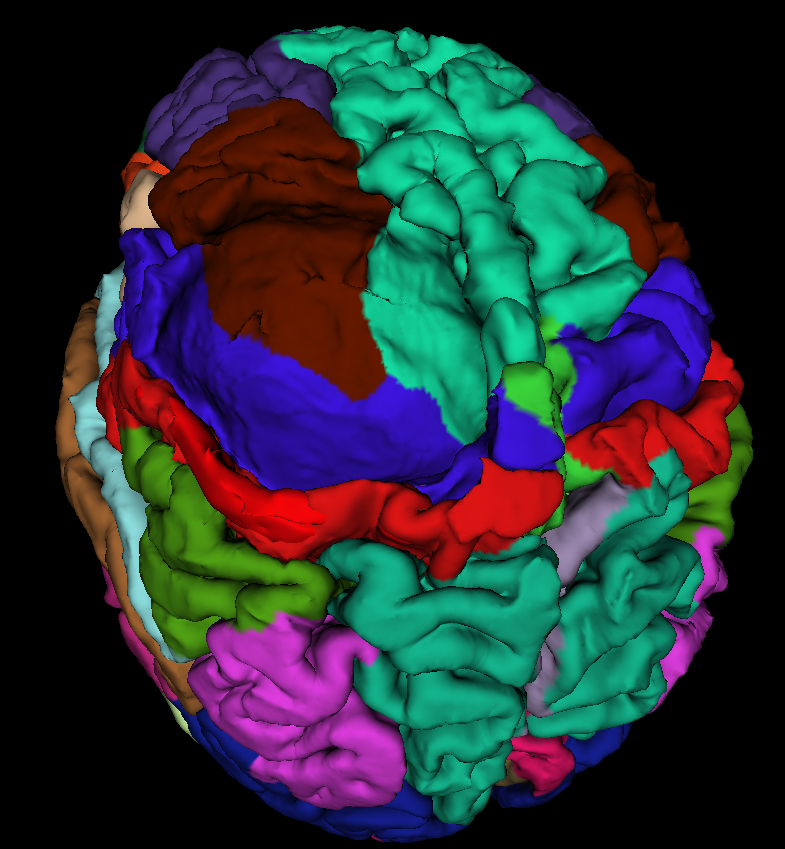

Supplement: S3 File — (ZIP) [file pone.0230754.s003.zip › SUB15/picture/color brain/1.jpg]

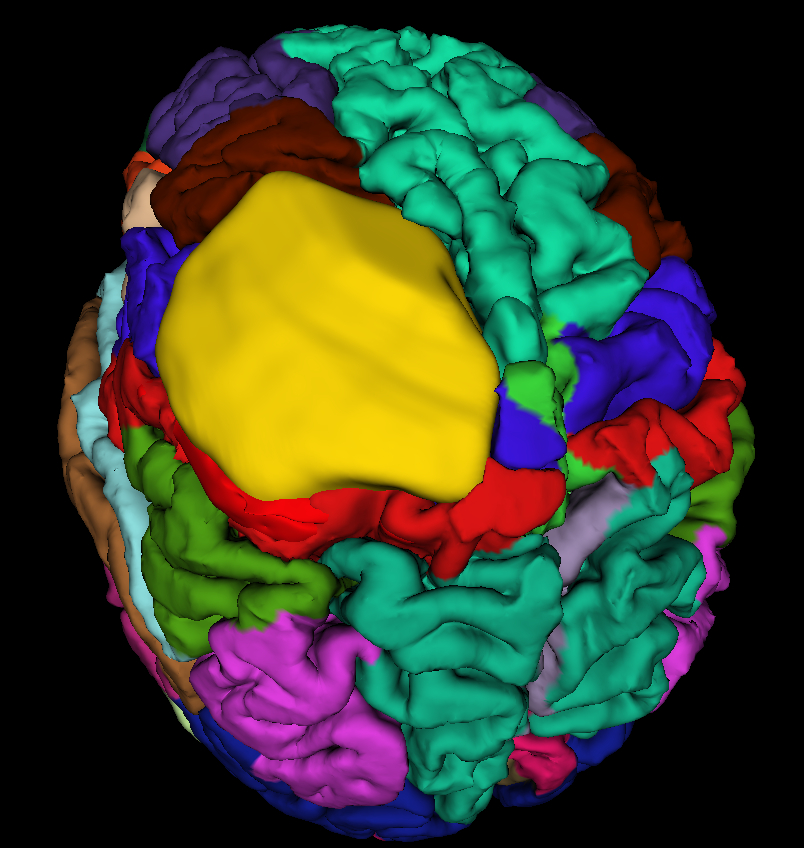

Supplement: S3 File — (ZIP) [file pone.0230754.s003.zip › SUB15/picture/color brain/2.jpg]

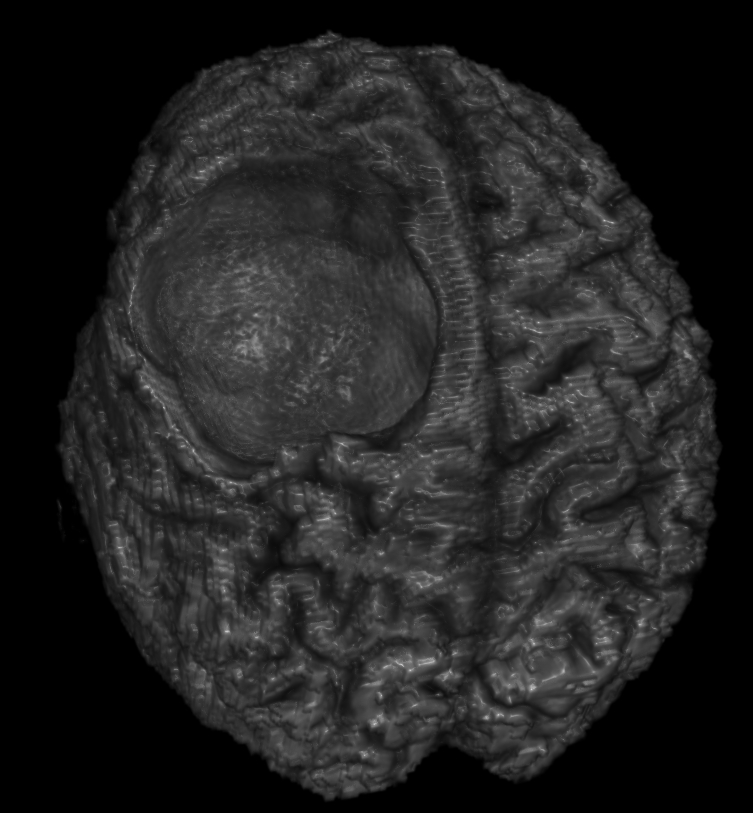

Supplement: S3 File — (ZIP) [file pone.0230754.s003.zip › SUB15/picture/VR/1.jpg]

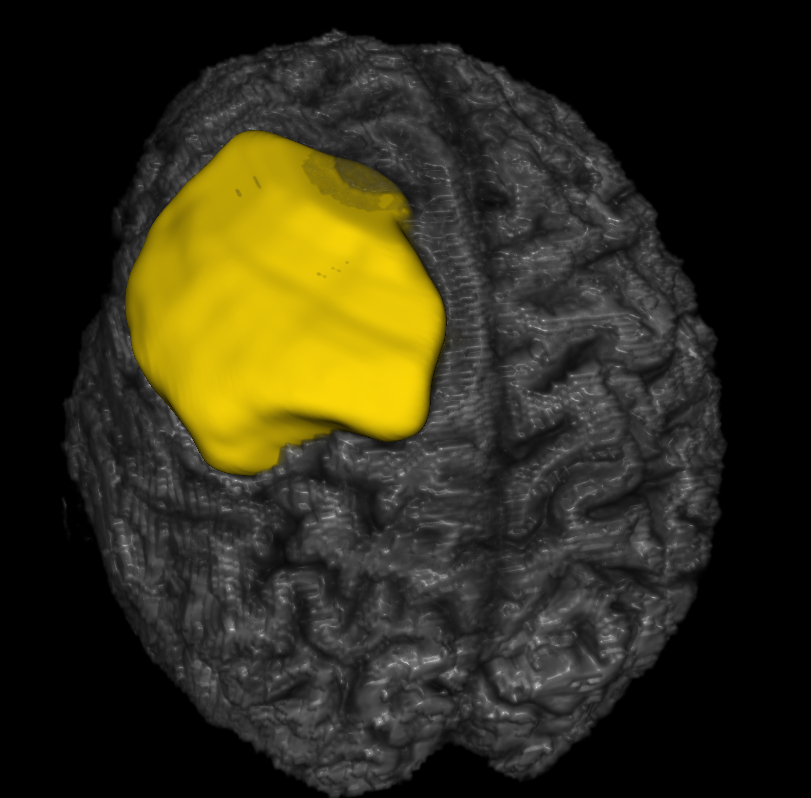

Supplement: S3 File — (ZIP) [file pone.0230754.s003.zip › SUB15/picture/VR/2.jpg]

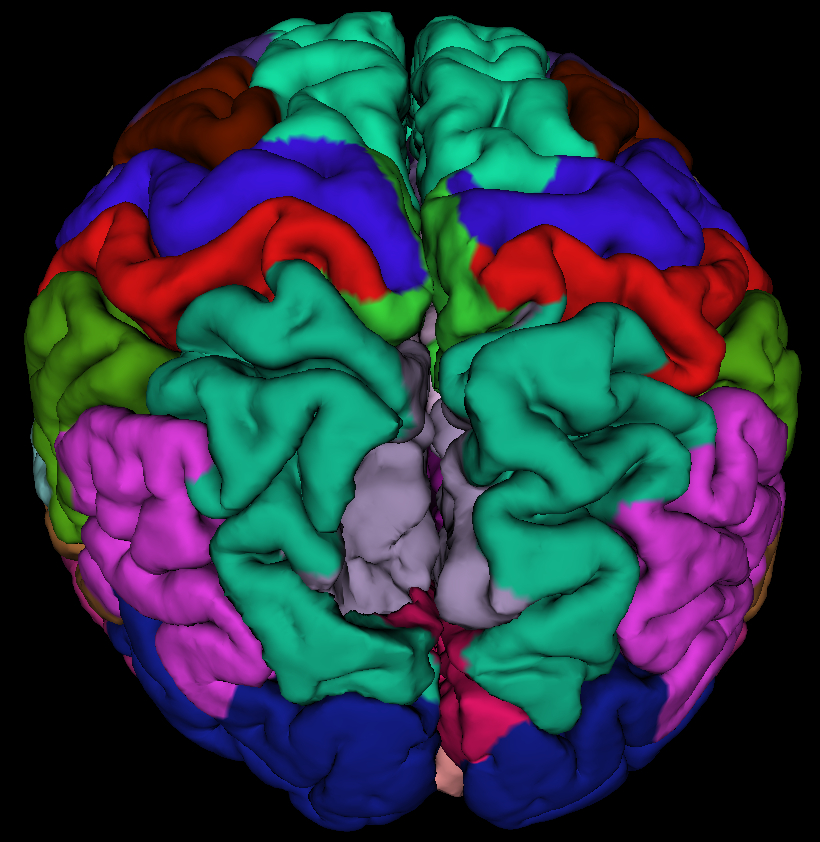

Supplement: S4 File — (ZIP) [file pone.0230754.s004.zip › SUB16/picture/color BRAIN/1.jpg]

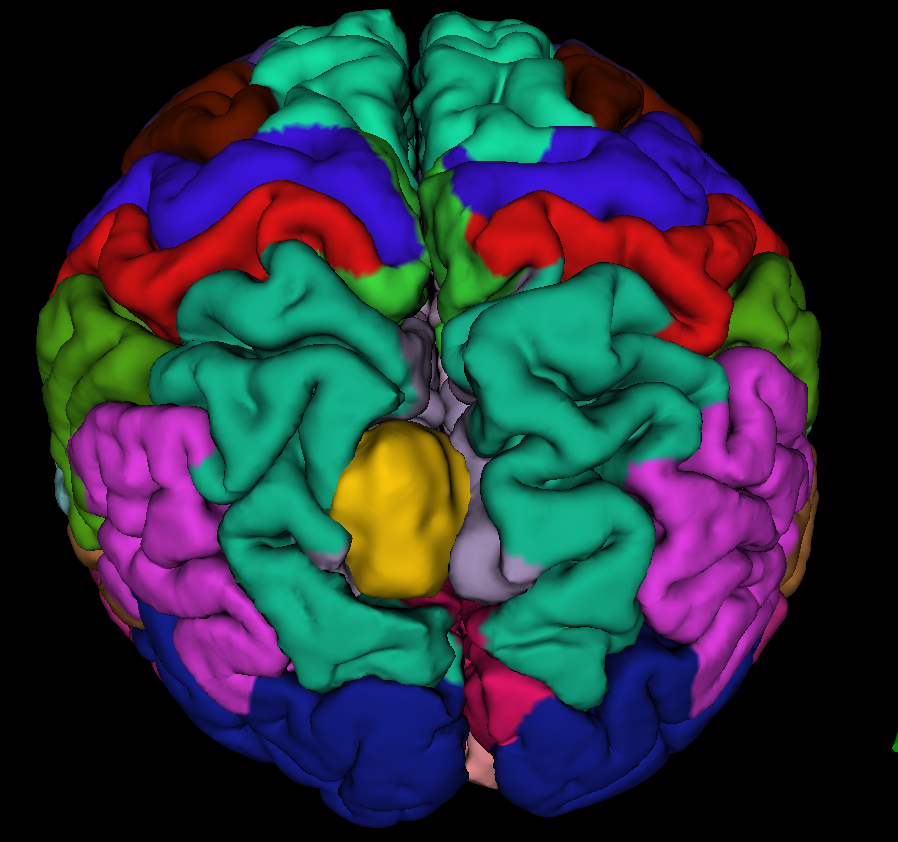

Supplement: S4 File — (ZIP) [file pone.0230754.s004.zip › SUB16/picture/color BRAIN/2.jpg]

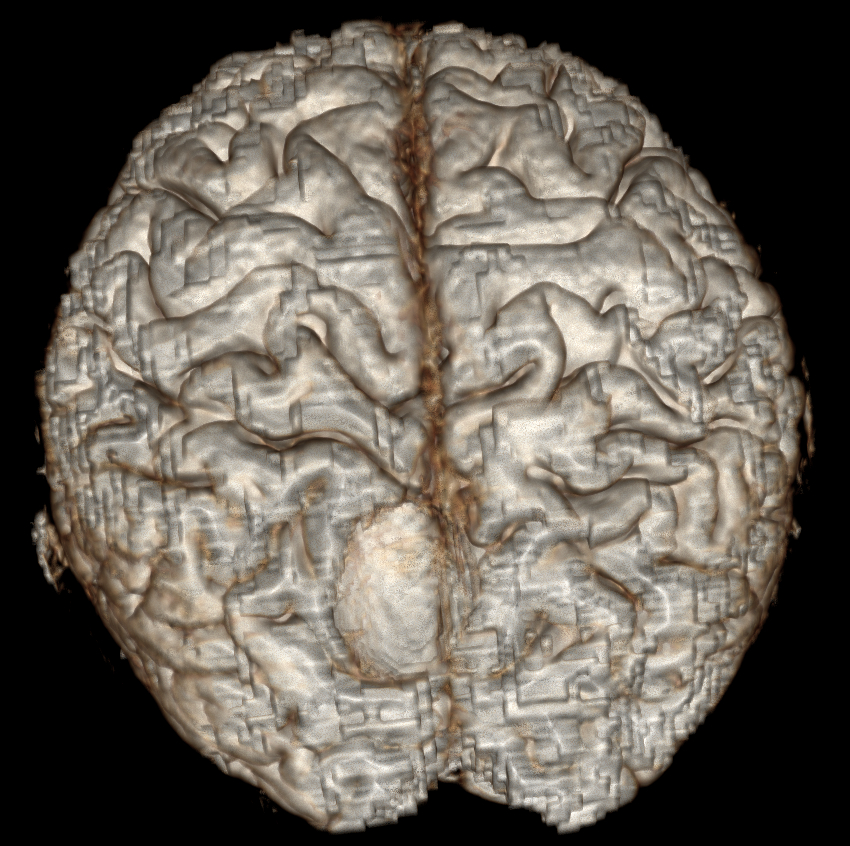

Supplement: S4 File — (ZIP) [file pone.0230754.s004.zip › SUB16/picture/VR/1.jpg]

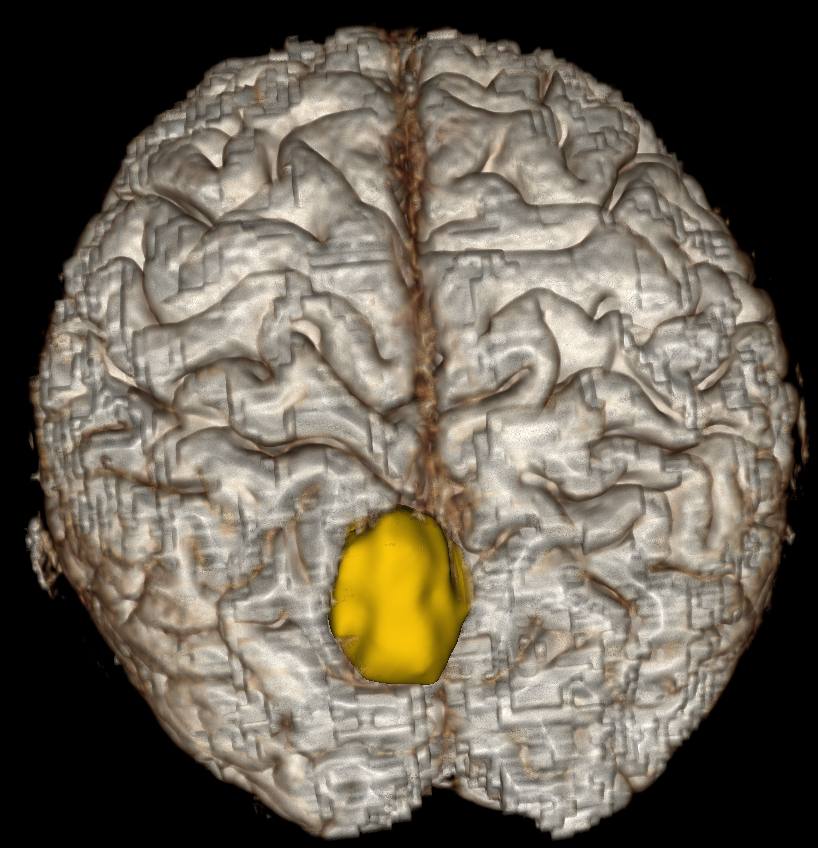

Supplement: S4 File — (ZIP) [file pone.0230754.s004.zip › SUB16/picture/VR/2.jpg]

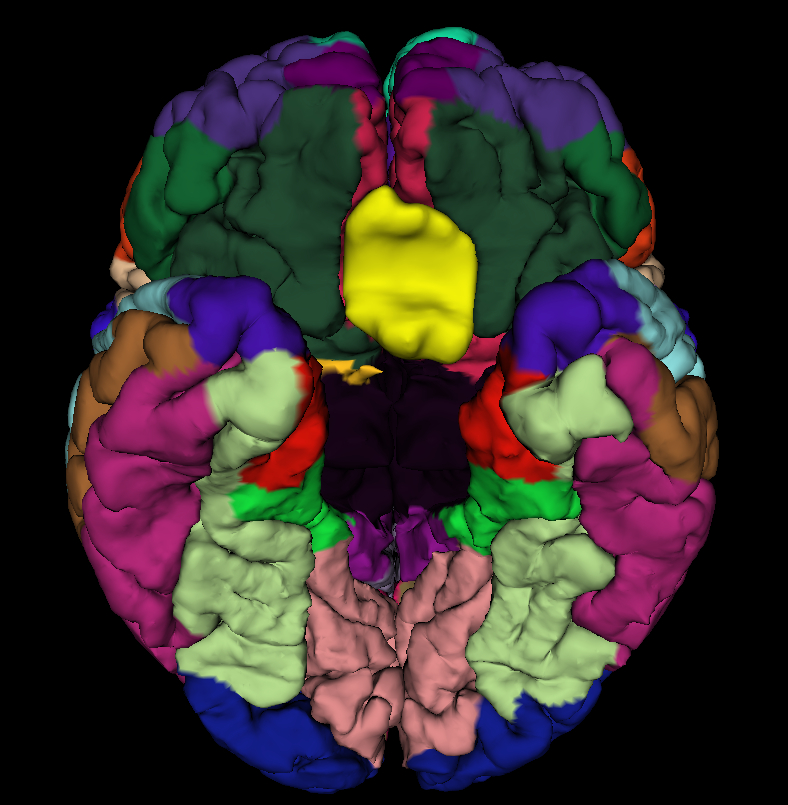

Supplement: S5 File — (ZIP) [file pone.0230754.s005.zip › SUB17/picture/color brain/1..jpg]

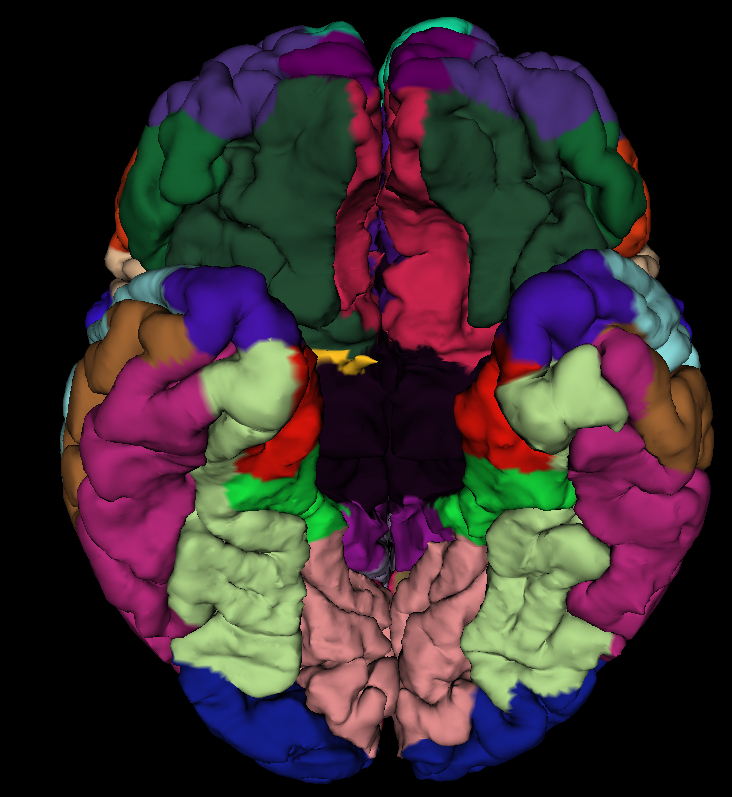

Supplement: S5 File — (ZIP) [file pone.0230754.s005.zip › SUB17/picture/color brain/2.jpg]

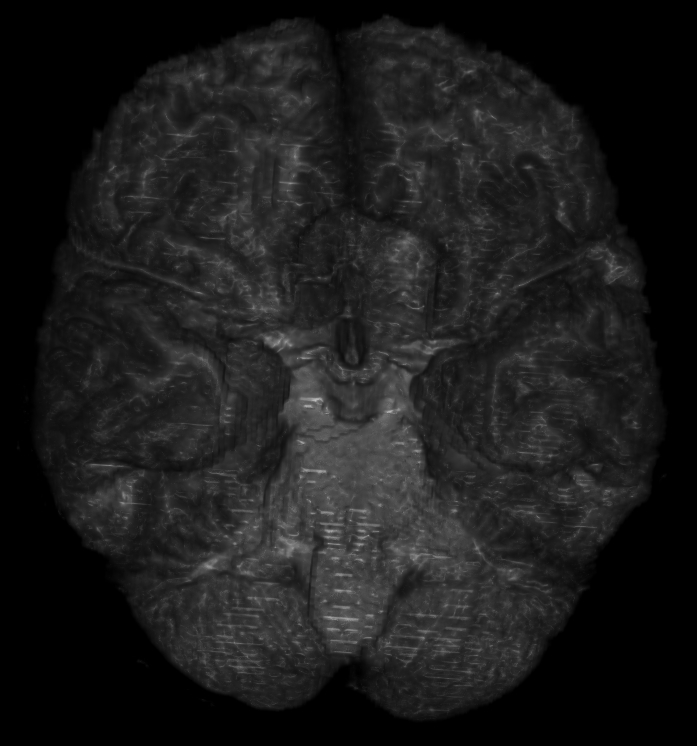

Supplement: S5 File — (ZIP) [file pone.0230754.s005.zip › SUB17/picture/VR/1.jpg]

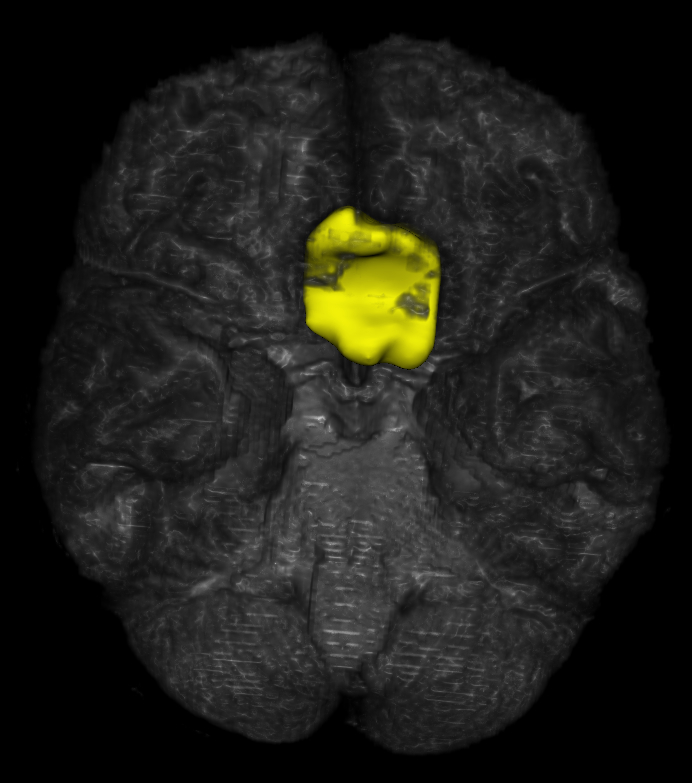

Supplement: S5 File — (ZIP) [file pone.0230754.s005.zip › SUB17/picture/VR/2.jpg]

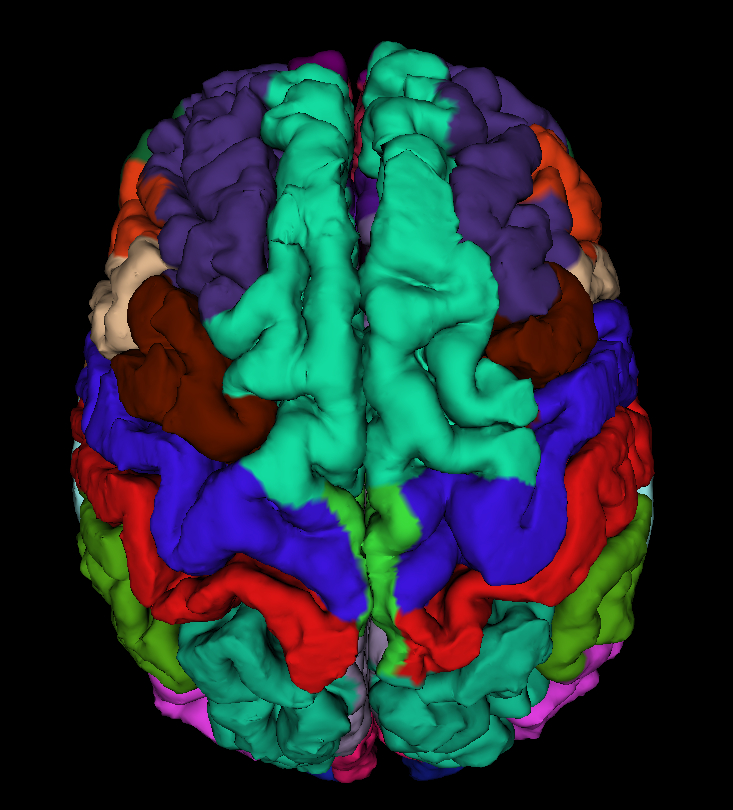

Supplement: S6 File — (ZIP) [file pone.0230754.s006.zip › sub24/picture/color brain/1.jpg]

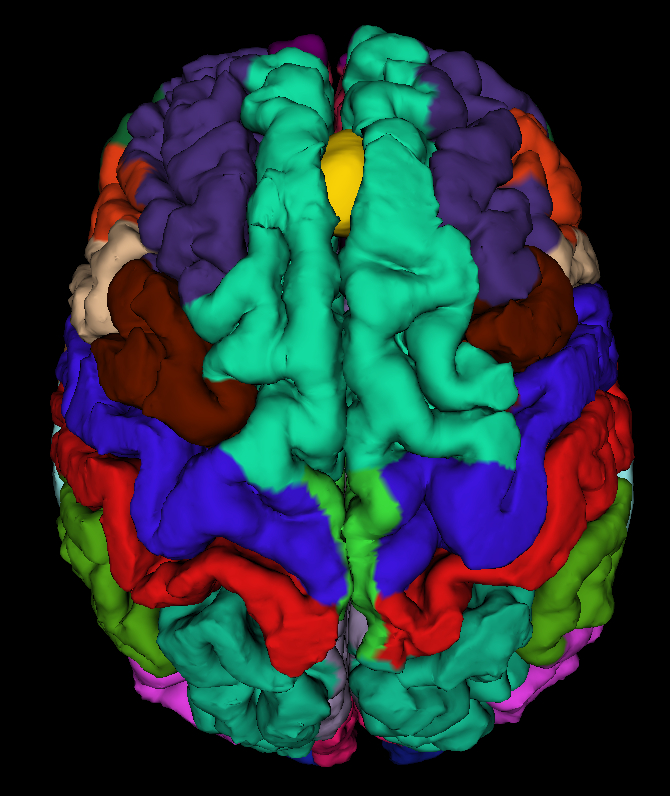

Supplement: S6 File — (ZIP) [file pone.0230754.s006.zip › sub24/picture/color brain/2.jpg]

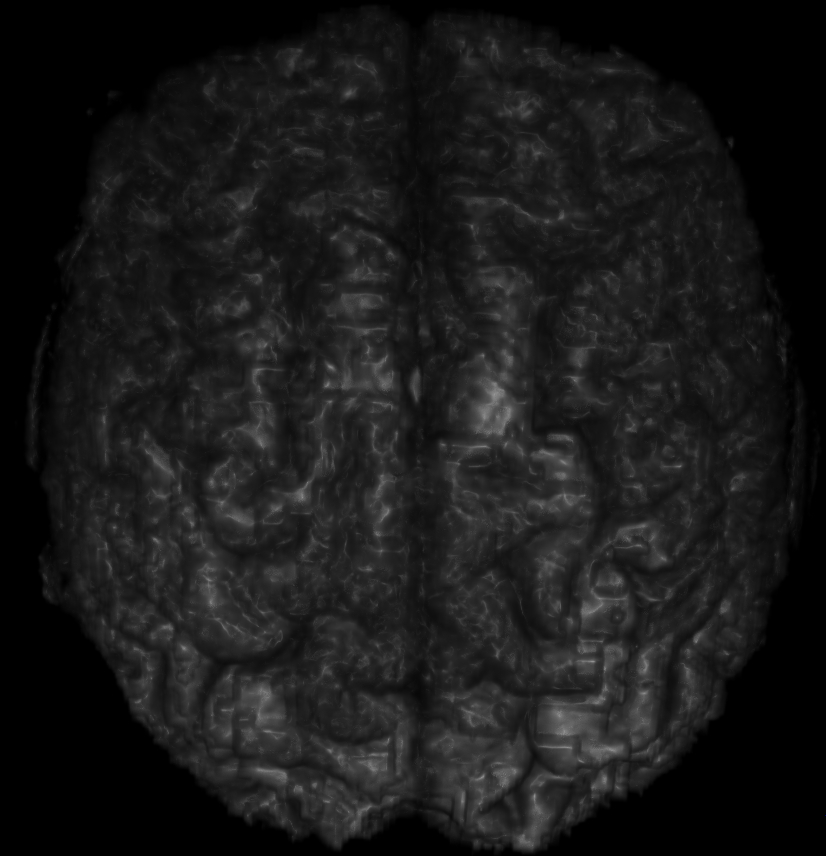

Supplement: S6 File — (ZIP) [file pone.0230754.s006.zip › sub24/picture/VR/1.jpg]

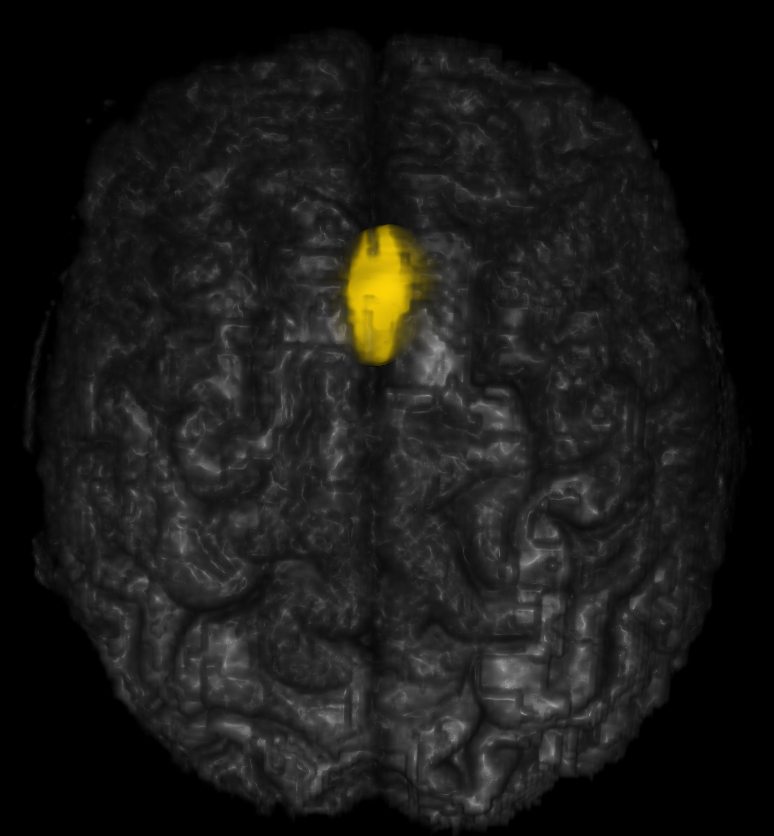

Supplement: S6 File — (ZIP) [file pone.0230754.s006.zip › sub24/picture/VR/2.jpg]
