# Supplementary material for: A new simple brain segmentation method for extracerebral intracranial tumors
Source: PLoS One. 2020 Apr 17;15(4):e0230754. doi: 10.1371/journal.pone.0230754 (PMC7164623; doi:10.1371/journal.pone.0230754)
Supplement: Attachment — Submitted filename: Response to Reviewers.docx [file pone.0230754.s007.docx]

Dear Editor,

We have studied the valuable comments from you, the assistant editor and reviewers carefully, and tried our best to revise the manuscript. We want to upload all the patient data, but because the Dicom/Nii data of all the patients is too large, we can only upload part of the patient data to verify the repeatability of our study. The point to point responds to the reviewer’s comments are listed as following:

Responds to the reviewer’s comments:

Reviewer 1

**Comment 1:** There is a lot of statistics hidden in the software that is not addressed in the methods. To my impression, these are pre-processed algorithms provided by the manufacturers, which are not clearly presented in the methods section, other than that they are being applied.

**Response:** According to the reviewer’s comment, we have added the software instructions, the algorithm of FreeSurfer is very complex; briefly, its processing includes motion correction and averaging of multiple volumetric T1-weighted images (when more than one is available), removal of nonbrain tissue using a hybrid watershed/surface deformation procedure, automated Talairach transformation, and segmentation of the subcortical white matter and deep gray matter volumetric structures. As a clinician, it is difficult to master the running algorithm of the software, and the official website does not mention too much. We were inspired by the simple calculation process provided by the official website. For the extracerebral intracranial tumors without serious peripheral edema, the boundary was clearly separated from the normal brain tissue. After the tumor was artificially labeled and removed, the interface between the normal brain tissue and the tumor is artificially divided. Then, the software could successfully carry out automatic brain tissue segmentation according to its original algorithm.

**Comment 2:** The manuscript in my opinion serves best the purpose of a methods paper. However, there is no other validation of the proposed method of inquiry other than the visual confirmation of realistic segmentation by the algorithm based on clinicians estimation.

**Response:** Thank you for your suggestion. We divided the definition of the sulci and gyri into three levels, the cerebral cortex model created by FreeSurfer and 3D volume-rendered images created by 3D Slicer were both evaluated simultaneously by 2 senior and 2 younger neurosurgeons, and scored according to subjective evaluation criteria. The two groups of scores were tested using a two-tailed Wilcoxon signed-rank test. SPSS was used for analysis, both senior and younger neurosurgeons agreed that the brain model created by FreeSurfer was better and had more easily identifiable substructures (p<0.01).

**Comment 3:** Throughout the paper there needs to be attention for the use of grammar and typos.

**Response:** Thank you for your careful work. We have submitted the original manuscript to the English translation company for revision.

**Comment 4:** The real valor of these segmentation algorithms would be in the application of pre-surgical planning for tumors with a lot of edema of intraparenchymal invasion. The manuscript would benefit with regard to scientific and translational impact if the authors could demonstrate any feasibility of these algorithms or the applied software to tackle these hurdles.

**Response:** Thank you for your advice. However, at present, there is no algorithm that can automatically segment the normal brain tissue from patients with brain tumors. Not just intracranial tumors cannot be segmented, but also extracranial tumors with severe peripheral edema also could not be segmented, because the blood brain barrier has been broken and gray matter boundary is not clear, the task of brain segmentation cannot be completed by Freesurfer software. We have tried to use Freesurfer software to segment the extracranial tumors with severe edema and intracranial tumors, [unfortunately](javascript:;), it can only get a defect model of brain tissue or even produce software errors that prevent brain segmentation. Therefore, the proposed method is currently only applicable to brain segmentation of extracranial tumors. Nevertheless, this method can still provide useful information for clinicians, especially when the extracranial tumor is located in a motor and language function areas.

Reviewer 2

**Comment 1:** Primarily, the data used to generate their figures is not made available, and as such their figures cannot be reproduced to validate their methodology.

**Response:** According to the reviewer’s comment, we want to upload all the patient data, but because the Dicom/Nii data of all the patients is too large, we can only upload part of the patient data to verify the repeatability of our study.

**Comment 2:** Secondarily, this article could benefit from a significant overhaul by a professional, English-language scientific editor. There are sections of the manuscript where the meaning of the authors is unclear, there are multiple incomplete sentences, and innumerable other grammatical and syntactical errors.

**Response:** Thank you for your careful work. We have submitted the original manuscript to the English translation company for revision.
