# Supplementary material for: A new simple brain segmentation method for extracerebral intracranial tumors
Source: PLoS One. 2020 Apr 17;15(4):e0230754. doi: 10.1371/journal.pone.0230754 (PMC7164623; doi:10.1371/journal.pone.0230754)
Supplement: Attachment — Submitted filename: Response to Reviewers.docx [file pone.0230754.s008.docx]

Dear Editor,

We have studied the valuable comments from you, the assistant editor and reviewers carefully, and tried our best to revise the manuscript. We have uploaded part of the patient data to verify the repeatability of our study.

The point to point responds to the reviewer’s comments are listed as following:

Responds to the reviewer’s comments:

Reviewer 3

**Comment 1:** The manuscript would benefit from revision by an English language editor.

**Response:** Thank you for your careful work. We have submitted the original manuscript to the English translation company for revision.

**Comment 2:** The authors have made a portion of their data available. The segmentation of the lesions appear accurate but there is no way to validate without the primary imaging data, etc.

**Response:** Thank you for your careful work. DICOM data is raw data, it contains the patient's privacy information. Therefore, we use dcm2nii software to transform DICOM data into “T1.nii” data, and remove the patient's privacy information. With the help of 3D Slicer，tumor was labeled and removed，the new data of “T1 without tumor. nii” is generated and can be loaded into Freesurfer software to segment the brain.

**Comment 3:** There is no validation of the method internally by the authors other than a relative neurosurgeon rating score, which is not a validated measure.

**Response:** Thank you for your advice. However, we are just trying to compare the difference of brain model that's been segmented by FreeSurfer software and traditional methods. The neurosurgeon rating score is the most direct and simple way to judge the pros and cons of the two models. In fact, the advantages of the former are obvious. Through literature review, there are similar evaluation methods, just like Karibe et al, in the early stage, used DWI to judge the damage degree of cerebral hemorrhage to corticospinal tract (CST), they also performed CST injury classification by visually observing the relationship between hematoma and CST. (Karibe H, Shimizu H, Tominaga T, et al. Diffusion-weighted magnetic resonance imaging in the early evaluation on of corticospinal tract injury to predict functional motor outcome in patients with deep intracerebral hemorrhage. J Neurosurg.2000;92(1):58-63.) Pandrangi et al. used patients' subjective scores to prove that VR technology is more advantageous in communicating with patients. (Pandrangi VC, Gaston B, Appelbaum NP, Albuquerque FC, Jr., Levy MM, Larson RA. The application of virtual reality in patient education. Ann Vasc Surg. 2019;59: 184-189.)

**Comment 4:** The argument that this technique can help identify function is only partially accurate. Neurophysiologcial monitoring and awake technique remain pillars of clinical care because anatomy does not always equal function. Receptive speech centers are a classic example. We know that clinically they are not restricted to angular gyrus and supramarginal gyrus but can be represented broadly in the inferior parietal lobule and posterior temporal lobe in the dominant hemisphere.

**Response:** Thank you for your advice. This is a mistake in writing. We have changed

the summary that the methods we have provided can be used by surgeons as a cheap and easy way to identify the “real” relationship between the extracerebral intracranial lesion and adjacent brain tissue before surgery and improve preoperative planning in any unit. Our method can only provide anatomical segmentation of brain tissue according to different brain structure templates.
